# GPx3-mediated redox signaling arrests the cell cycle and acts as a tumor suppressor in lung cancer cell lines

**DOI:** 10.1371/journal.pone.0204170

**Published:** 2018-09-27

**Authors:** Byung Chull An, Yoo-Duk Choi, In-Jae Oh, Ju Han Kim, Jae-Il Park, Seung-won Lee

**Affiliations:** 1 Department of Anatomy, Chonnam National University Medical School, Hwasun-eup, Hwasun-gun, Jeollanam-do, Korea; 2 Department of Pathology, Chonnam National University Hospital, Dong-gu, Gwangju, Korea; 3 Department of Lung and Esophageal Cancer Clinic, Chonnam National University Hwasun Hospital, Hwasun-eup, Hwasun-gun, Jeollanam-do, Korea; 4 Department of Internal Medicine, Chonnam National University Hospital, Dong-gu, Gwangju, Korea; 5 Animal Facility of Aging Science, Korea Basic Science Institute, Buk-gu, Gwangju, Korea; University of South Alabama Mitchell Cancer Institute, UNITED STATES

## Abstract

Glutathione peroxidase 3 (GPx3), a major scavenger of reactive oxygen species (ROS) in plasma, acts as a redox signal modulator. However, the mechanism underlying GPx3-mediated suppression of cancer cell growth is unclear. The aim of this study was to identify these mechanisms with respect to lung cancer. To enhance the redox modulating properties of GPx3, lung cancer cells were subjected to serum starvation for 12 h, resulting in ROS generation in the absence of oxidant treatment. We then investigated whether suppression of tumorigenesis under conditions of oxidative stress was dependent on GPx3. The results showed that GPx3 effectively suppressed proliferation, migration, and invasion of lung cancer cells under oxidative stress. In addition, GPx3 expression led to a significant reduction in ROS production by cancer cells and induced G2/M phase arrest. We also found that inactivation of cyclin B1 significantly suppressed by nuclear factor-κB(NF-κB) inactivation in lung cancer cells was dependent on GPx3 expression. To further elucidate the mechanism(s) underlying GPx3-medited suppression of tumor proliferation, we next examined the effect of GPx3-mediated redox signaling on the ROS-MKP3-extracellular signal-regulated kinase (Erk)-NF-κB-cyclin B1 pathway and found that GPx3 strongly suppressed activation of the Erk-NF-κB-cyclin B1 signaling cascade by protecting MKP3 (an Erk-specific phosphatase) from the effects of ROS. Thus, this study demonstrates for the first time that the GPx3 suppresses proliferation of lung cancer cells by modulating redox-mediated signals.

## Introduction

Homeostasis of the cellular redox environment is maintained by a balance between ROS production and ROS scavenging, which is controlled by antioxidant enzymes. For example, superoxide dismutase enzymes (MnSOD, CuZnSOD, and Ec-SOD) catalyze the conversion of superoxide anions (O_2_^∙-^) to hydrogen peroxide (H_2_O_2_). Catalase (CAT), peroxiredoxin (Prx), and glutathione peroxidase (GPx) then convert H_2_O_2_ to water.

ROS are classically considered toxic to cells and as such are implicated in the pathogenesis of many diseases, although they are endogenously generated in cells. ROS damage important cellular components such as proteins, DNA, and membrane lipids, which can result in cell death. However, recent studies demonstrate that ROS also act as a second messenger to modulate mitogenic signal transduction in various mammalian cells [1]. Furthermore, ROS play roles in various physiological and pathological processes, including cell proliferation, adhesion, and survival [2]. ROS-induced DNA damage disrupts genomic integrity and is an important cause of cancer in humans [3]. Malignant cells produce more ROS than normal cells [4]. Importantly, levels of ROS scavenging enzymes such as SODs, GPxs, and Prxs are significantly altered in cancer cells [5, 6]. These essential redox regulating antioxidant enzymes play an extremely important role: SODs catalyze the conversion of O_2_^∙-^ into H_2_O_2_, which is then converted to O_2_ and H_2_O by peroxidases and catalase [7].

Many types of cancer cell exhibit lower expression of antioxidant enzymes, especially MnSOD, than their normal counterparts [7]. Numerous studies demonstrate that overexpression of MnSOD in tumor cells inhibits carcinogenesis [8], suggesting that MnSOD acts as a tumor suppressor. For example, MnSOD regulates a ROS switch that favors a superoxide signal that regulates the proliferative cycle, and a H_2_O_2_ signal that supports quiescent growth. Higher levels of MnSOD activity are associated with quiescence, whereas lower levels support proliferation. MnSOD activity–regulated transition between quiescent and proliferative growth is associated with changes in expression of cyclin D1 and cyclin B1 [9]. Taken together, these findings support the hypothesis that MnSOD activity maintains the redox balance and a normal chronologic life span. MnSOD also negatively regulates NF-κB expression/activity by deactivating ROS [10]. The first intron of the human cyclin B1 gene harbors an NF-κB binding site, as evidenced by the finding that MnSOD-mediated downregulation of NF-κB negatively regulates cyclin B1 expression in MCF-7 breast cancer cells [11]. Thus, SOD enzymes play a key role in redox regulation and diverse cellular functions.

CAT efficiently catalyzes conversion of H_2_O_2_ to water and O_2_. Moreover, it degrades peroxynitrite (ONOO^−^) via an enzymatic reaction [12]. Reduced CAT activity has been reported in cancer [13] and suppression of CAT increases H_2_O_2_ levels, which in turn stimulates H_2_O_2_-dependent signaling pathways that promote tumor progression [14]. Thus, CAT might also modulate H_2_O_2_- and NO/ONOO^−^-mediated signaling pathways. NADPH oxidase (NOX) increases O_2_^∙-^ levels in cancer cells [12, 15]; therefore, malignant cells may acquire a membrane-associated catalase. Various studies show that overexpression of membrane-associated catalase on the surface of tumor cells protects them against apoptosis induced by intercellular ROS signaling [12, 15]. Efficient protection of tumor cells by membrane-associated catalase does not contradict the finding that, in general, tumor cells express lower levels of catalase than normal tissues; this is because the surface of the tumor cell (with its high local concentration of catalase) represents only a small proportion of the total cellular mass within a tumor [16].

Prxs are thioredoxin dependent peroxidases that catalyze the reduction of H_2_O_2_, organic hydroperoxides, and peroxynitrite to balance intracellular ROS levels [17]. Recent studies report that Prxs (type 1–4) regulate cell signaling by directly interacting with specific redox-sensitive proteins or by deactivating ROS generated by NOX at the cell membrane [18]. For example, Prx1 suppresses oncogene-induced cell transformation by protecting PTEN/Akt from ROS [19]. Endothelial Prx2 is a proangiogenic factor that functions by protecting vascular endothelial growth factor receptor 2 (VEGFR2) from oxidative inactivation [20]. Prx-mediated maintenance of the redox balance regulates diverse cellular processes, including proliferation, migration, apoptosis, and metabolism [18]. A major antioxidant family comprising eight different GPx isoenzymes (GPx1-8) catalyzes the degradation of H_2_O_2_, organic hydroperoxide, and lipid peroxides by reduced glutathione [21]. Five different GPx isoenzymes (GPx1-4, 6), all containing selenocysteine, are present in humans and all exhibit tissue-specific expression and different substrate specificities [22]. The antioxidative properties of overexpressed GPx family proteins protect cells against oxidative damage [23]. For example, GPx1 protects both FL5 and MDBK cells from H_2_O_2-_mediated apoptosis [24]. The levels of GPx1 protein in the cytoplasm of human pancreatic carcinoma cells are lower than those in normal pancreatic cells [25]; moreover, GPx activity in pancreatic cancer cell lines is lower than that in normal pancreatic cell lines. Silencing of antioxidant enzymes may also trigger malignant transformation. Plasma GPx (GPx3) is the most important selenoenzyme involved in deactivating ROS, acting via thioredoxin and glutathione as reduction co-substrates [21, 26]. Recently, we demonstrated glucocorticoid receptor-mediated upregulation of GPx3 in lung cancer cells [27]. Downregulation of GPx3 via promoter hypermethylation occurs in many types of human cancer, suggesting that GPx3 acts as a tumor suppressor [28, 29]. Reduced expression of GPx3 might impair defense against ROS, resulting in mutation of genes involved in carcinogenesis [30]. Loss of GPx3-mediated activity may be associated with the early stages of inflammation-mediated carcinogenesis [31].

Recently, we have performed a study to discover a non-small-cell lung cancer (NSCLC) biomarker in plasma from patients. The differentially expressed genes (DEGs), derived from the analysis of 9 transcriptome datasets (from public databases), were double-checked for detectability in the secretome of six lung cancer cell lines, in the pool of a certain patient plasma and finally in the current platform of multiple reaction monitoring (MRM) and immunodetection assay. We have got four candidate biomarkers passing through all the steps, only two (GPx3 and BCHE) of which were demonstrated to statistically significant difference in the blood level between NSCL and Control. According the result of this study, GPx3 expression in lung cancer patients is lower, both in the cells (or tissue) of patient lungs and in the patient plasmas, than that in healthy controls. This shows how GPx3 distribute throughout the lung tissue and the blood of patients, which implies this molecule might serve as tumor suppressor [32]. Thus, GPx3 exerts tumor suppressor activity by directly or indirectly regulating cell growth and proliferation via an as yet unknown mechanism(s) [29, 30, 33]. Therefore, to examine the antioxidant-mediated tumor suppressive function of GPx3, we subjected lung cancer cells to serum starvation, which stimulates ROS production without the need for oxidant treatment [34–36].

The main purpose of the present study was to examine the mechanism(s) underlying GPx3-mediated tumor suppression in lung cancer cells. We also ought to determine the molecular species responsible for this tumor suppressive activity. We hypothesized that silencing GPx3 would induce tumorigenesis in lung cancer cells. We found that overexpression of GPx3 significantly inhibited the proliferation, migration, and invasion of lung cancer cells and arrested growth at the G2/M phase. We also found that expression of cyclin B1 (a downstream modulator of cell cycle arrest) was specifically reduced upon expression of GPx3. GPx3 may achieve this by regulating the MKP3-Erk-NF-κB-cyclin B1 signal cascade by deactivating ROS. Thus, this is the first study to show that a GPx3-mediated redox signaling pathway suppresses proliferation of lung cancer cells.

## Materials and methods

### Reagents and antibodies

Human recombinant GPx3 (hGPx3) was purchased from Enzo Life Sciences (MI, USA). Antibodies specific for GPx3, MKP3, Erk, NF-κB, cyclinB1, lamin B1, and actin were purchased from Santa Cruz Biotechnology (CA, USA) and Cell Signaling Technology (MA, USA). PDTC (pyrrolidine dithiocarbamate) was purchased from Sigma (CA, USA).

### Cell culture

H157, H460, A549, H1299, H1650, and H1975 lung cancer cells (ATCC, Manassas, VA) were cultured in RPMI 1640 medium (Gibco, Los Angeles, CA, USA) supplemented with 10% fetal bovine serum (FBS) (Gibco), 100 U/ml penicillin, and 100 U/ml streptomycin (Invitrogen, Carlsbad, CA, USA) at 37°C/5% CO_2_ [37]. To induce oxidative stress, lung cancer cells were exposed to RPMI 1640 without FBS for 12 h before being allowed to recover for 6 h in RPMI 1640 containing 10% FBS [35, 36].

### Plasmid construction

The human *GPx3* gene was amplified from lung cancer cell cDNA by PCR using the following primers: *GPx3*-F (XbaI), 5’- tctagagacacctcagacgga-3’ and *GPx3*-R (SalI), 5’-gtcgaccttcagttacttcctc-3’. The PCR reaction mixture comprised ApONEtm Taq premix (GeneAll, Seoul, Korea), 2 μl of cDNA, and 20 pmol of each primer (total volume, 20 μl). The PCR conditions were as follows: denaturation at 95°C for 5 min, followed by 38 cycles of denaturation at 95°C for 30 sec, annealing at 58°C for 30 sec, and extension at 72°C for 1 min, followed by a final extension step at 72°C for 10 min. The amplified *GPx3* DNA fragment (771 bp) was digested with XbaI/SalI and cloned into the pCI-neo-SECIS (NotI) vector via the XbaI/SalI site (Promega, Madison, WI, USA). The construct was then transformed into *Escherichia coli* DH5α for amplification. All restriction enzymes were purchased from New England BioLabs (NEB, Ipswich, MA, USA).

### Reverse transcription polymerase chain reaction (RT-PCR)

Total RNA (1 μg) from lung cancer cells was reverse transcribed to complementary DNA (cDNA) using hyperscript^tm^ RT premix (with oligo dTs) (GeneAll) in a final volume of 20 μl. This mixture was incubated for 1 h at 55°C and then heated to 95°C for 10 min to inactivate the RT. The resulting cDNAs were used to amplify the following specific targets by PCR: *GPx3*, *cyclin B1*, and *β-actin*. Primers in Table 1 were designed such that any genomic DNA product could be distinguished from the target cDNA by size. The PCR reactions comprised ApONEtm Taq premix (GeneAll), 2 μl of cDNA, and 20 pmol of each primer (total volume, 20 μl). The PCR conditions were as follows: denaturation at 95°C for 10 minutes, followed by 37 cycles of denaturation at 95°C for 1 minute, annealing at 58°C for 1 min, and extension at 72°C for 1 min, followed by a final extension step at 72°C for 10 min.

**Table 1:** Primers used in this study.

Name	Sequences (5’ to 3’)
***GPX3*-F**	TGGTCATTCTGGGCTTTCCC
***GPX3*-R**	CCAGAAGAGGCGGTCAGATG
***Cyclin B1*-F**	AAGAGCTTTAAACTTTGGTCTGGG
***Cyclin B1*-F**	CTTTGTAAGTCCTTGATTTACCATG
***Actin*-F**	GGACTTCGAGCAAGAGATGG
***Actin*-R**	AGCACTGTGTTGGCGTCAAG

### Western blot analysis

Following the indicated treatments, cells were washed in phosphate-buffered saline (PBS) and lysed in ice-cold RIPA buffer [20 mM Tris (pH 7.5), 150 mM NaCl, 1 mM EDTA, 0.5% sodium deoxycholate, 1% Igepal CA-630, and 0.1% sodium dodecyl sulfate] supplemented with protease inhibitor cocktail (Sigma). The cell lysates were resolved in 4–20% Tris-glycine Ready gels (Bio-Rad) and proteins transferred to PVDF membranes. Subsequently, the membranes were blocked with 5% nonfat dry milk in TBS-T buffer [20 mM Tris-HCl (pH 7.5), 150 mM NaCl and 0.01% Tween 20] and then probed with primary antibodies specific for GPx3, MKP3, Erk, NF-κB, cyclinB1, lamin B1, and actin, all diluted in TBS-T buffer containing 5% non-fat milk. The membranes were then incubated with the appropriate horseradish peroxidase-conjugated secondary antibodies (Santa Cruz Biotechnology) and reactive bands visualized by chemiluminescence (GE Healthcare, Piscataway, NJ).

### Cell transfection

Lung cancer cell lines were transfected with GPx3 shRNA (Santa Cruz Biotechnology) or plasmid DNA (pCI-neo-SECIS and pCI-neo-SECIS::*GPx3*, labmade). The day before transfection, lung cancer cells were plated in 6-well plates at a density of 7×10^5^ cells per well. After incubating overnight, cells were transfected using Lipofectamine 3000 (Invitrogen) in accordance with the manufacturer’s instructions [30, 33]. The transfected cells were selected in RPMI 1640 containing antibiotics.

### Cell proliferation assay

After induction of oxidative stress through serum starvation, lung cancer cells were exposed to 0.05% trypsin and reseeded in 96-well plates at a density of 2000 cells per well in RPMI 1640 containing 10% FBS. Treatments were initiated after the cells had attached to the wells. At the appropriate time points (every 12 h), cell viability was measured in a MTT assay. Briefly, 10 μl of MTT (5 mg/ml in PBS) was added to each well, followed by incubation at 37°C for 4 h. The formazan crystal sediments were then dissolved in 100 μl of dimethyl sulfoxide and absorbance measured at 570 nm in a microplate reader (Bio-Rad, Hercules, CA). Each treatment was performed in six replicate wells.

### Scratch wound healing assay

After being subjected to oxidative stress, lung cancer cells were trypsinized, resuspended in RPMI 1640 containing 10% FBS and reseeded in 96 well plates at a density that yielded 100% confluence. To examine the role of GPx3 in cell migration, a scratch was made across each well using a pipette tip. Cells were then cultured in complete medium and images taken for 72 h to monitor wound healing.

### Matrigel invasion assay

After being subjected to oxidative stress, lung cancer cells were trypsinized and reseeded into the upper chamber of a Transwell (BD Biosciences) at a density of around 5×10^4^ cells/0.5 ml of serum-free DMEM. The lower chamber was then filled with RPMI 1640 containing 10% FBS as a chemo attractant. The cells were incubated at 37°C/5% CO_2_ for 72 h. The cells remaining on the upper surface of the chamber were removed using a cotton tip. Cells that had penetrated through the membrane were fixed in methanol at -20°Cfor 20 min and counted under a light microscope. The experiment was performed in triplicate.

### Cell cycle analysis

After being subjected to oxidative stress, cells were trypsinized and harvested in phenol-red-free medium supplemented with charcoal-stripped FBS. Cells (1 × 10^6^) were washed and suspended in 500 μl PBS, fixed with ice-cold 100% ethanol (added dropwise with constant agitation), and incubated on ice for 20 min. A cell pellet was obtained by centrifugation, suspended in 500 μl of propidium iodide/RNase solution, incubated in the dark at room temperature for 20 min, washed, and analyzed using a FACSCalibur (BD Biosciences) cytometer equipped with Diva software (BD Biosciences). A total of 10^4^ events were acquired per sample. The distribution of cells at each phase of the cell cycle was determined using the CycleTEST PLUS DNA Reagent Kit (BD Biosciences). Three distinct phases could be recognized in a proliferating cell population: G1, S (DNA synthesis phase), and G2.

### Fluorometric detection of H_2_O_2_ production

After being subjected to oxidative stress, the levels of H_2_O_2_ and ROS were measured using the Amplex UltraRed hydrogen peroxide assay kit (Invitrogen) and H2DCFDA, respectively. To measure ROS levels, cells were incubated with 5 μM H2DCFDA for 30 min and fluorescence intensity measured in a microplate reader (Bio-Rad, Hercules, CA) at 485/535 nm, according to the manufacturer's protocol. For H_2_O_2,_ florescence intensity was measured at 530/590 nm. Relative ROS levels were calculated in terms of arbitrary fluorescence units.

## Results

### Lung cancer cell lines show altered expression of GPx3

**Fig 1:**
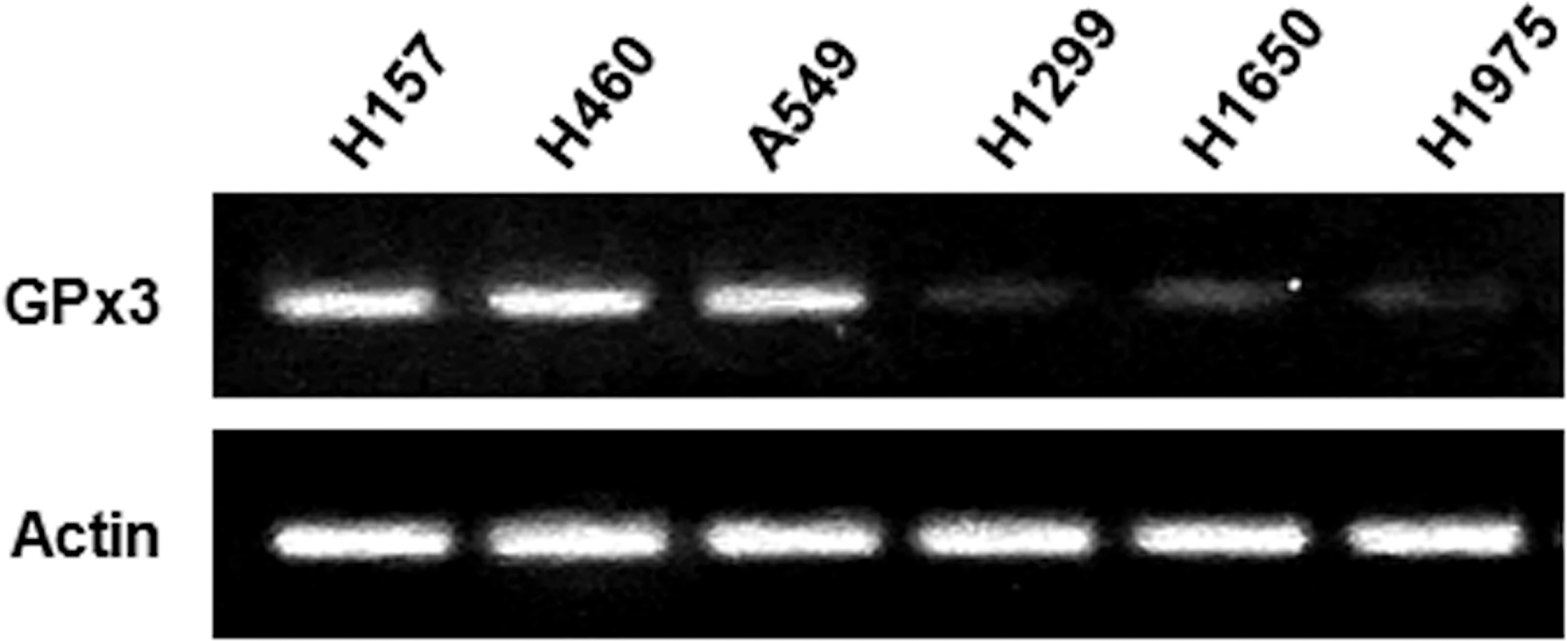
Altered GPx3 expression in lung cancer cells. Expression of GPx3 mRNA in H157, H460, A549, H1299, H1650, and H1975 cell lines was examined by RT-PCR. Actin was used as an internal control for data normalization.

We asked whether altered GPx3 expression is a common occurrence in lung cancer cell lines. We measured expression of GPx3 mRNA in six cell lines (H157, H460, A549, H1299, H650, and H1975) by RT-PCR and found high expression in H157, H460, and A549 cells; however, H1299, H1650, and H1975 cells exhibited low levels of GPx3 (Fig 1). These different levels of GPx3 expression might be due to differences in the methylation [38, 39] and/or glucocorticoid receptor-mediated regulation of the GPx3 promoter [27].

### Tumor suppressor activity of GPx3 in lung cancer cell lines

**Fig 2:**
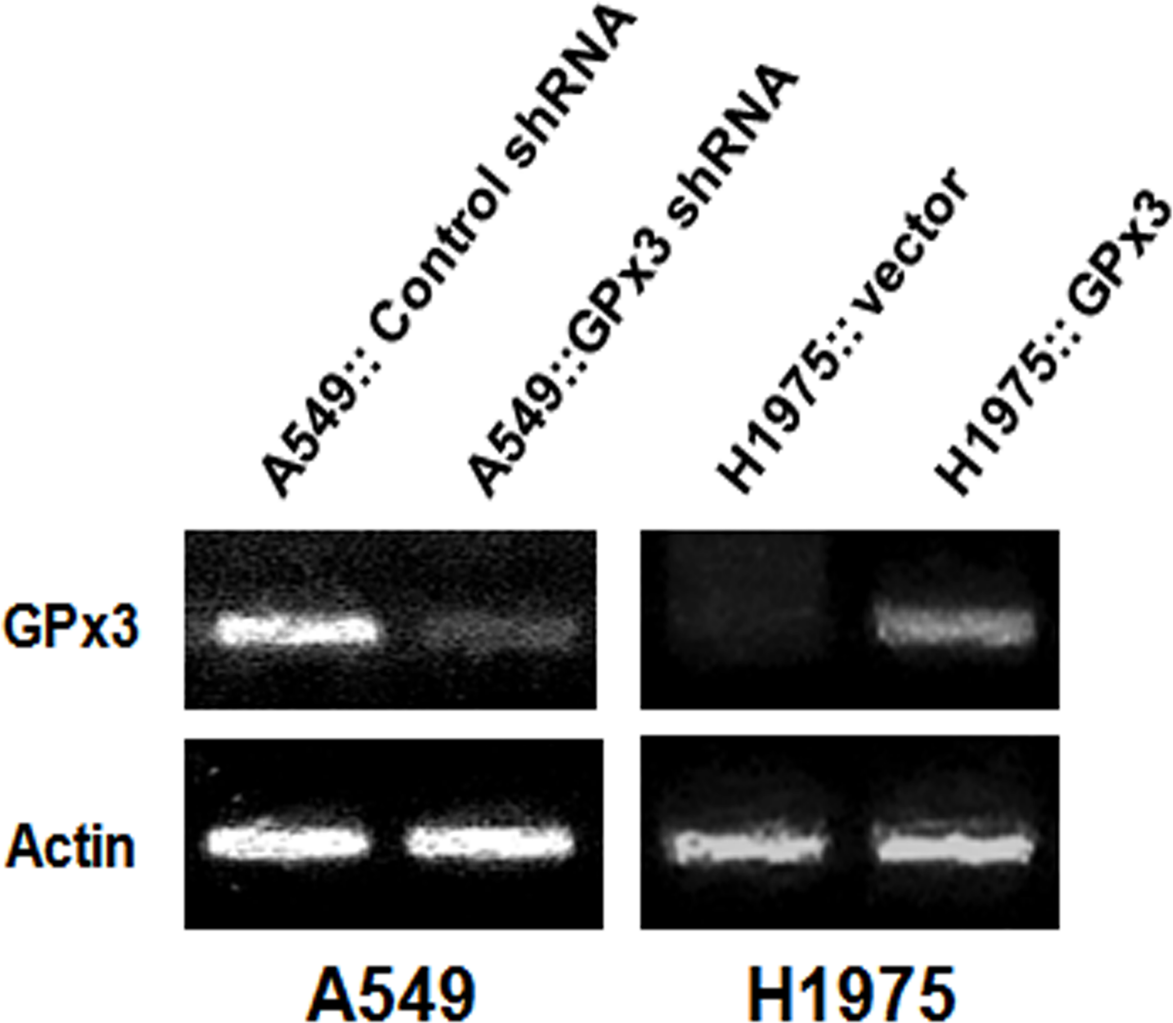
Overexpression and knockdown of GPx3 expression in lung cancer cells. GPx3 shRNA(shGPx3) was used to knockdown endogenous GPx3 in A549 (with high constitutive expression of GPx3), whereas a mammalian expression vector (pCI-neo-SECIS) was used to over-express GPx3(GPx3) in H1975 cells (that show low constitutive expression of GPx3). Data were normalized against those from cells transfected with control shRNA (shCON) or empty vector (EV).

**Fig 3:**
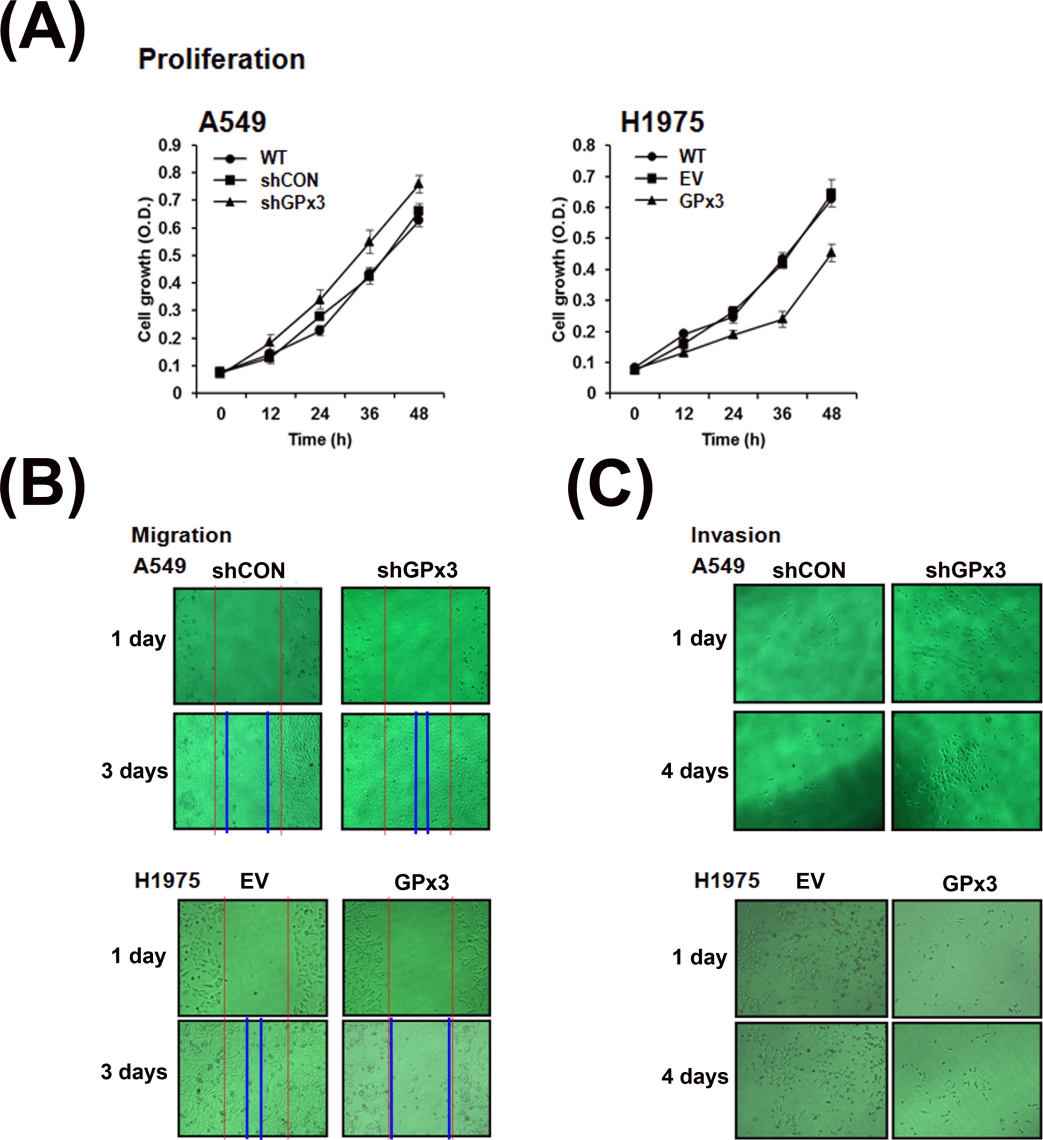
Tumor suppressive effects of GPx3 in lung cancer cells subjected to oxidative stress. Effect of GPx3 expression on lung cancer cell proliferation (A), migration (B), and invasion (C). GPx3 shRNA(shGPx3) was used to knockdown endogenous GPx3 in A549 (with high constitutive GPx3 expression), whereas a mammalian expression vector (pCI-neo-SECIS) was used to over-express GPx3(GPx3) in H1975 cells (with low constitutive GPx3 expression). Untransfected cells (WT) were used as a negative control. Cell proliferation was observed over a period of 2 days, whereas cell migration and invasion were observed over a period of 3–4 days. Data are representative of three independent experiments.

The above data showed that A549 cells expressed higher levels of GPx3 than H1975 cells. Therefore, to examine whether GPx3 suppresses tumor growth, we generated H1975 cells overexpressing GPx3 and knocked down GPx3 expression in A549 cells using shRNA (Fig 2). We then compared each of these selected stable clones with the parental cell lines and verified altered expression level of GPx3 (either overexpression or reduced expression) in each cell line. To examine whether the tumor suppressive function of GPx3 is associated with its antioxidant activity, we subjected cells to serum starvation to induce oxidative stress. After starvation for 12 h, the lung cancer cell lines were subjected to proliferation, scratch wound migration, and invasion assays (Fig 3). The results revealed that overexpression of GPx3 efficiently suppressed proliferation (Fig 3A), healing (Fig 3B), and invasion (Fig 3C) of tumor cells, whereas knocking down GPx3 had the opposite effect.

### Silencing of GPx3 increases ROS production by lung cancer cells

**Fig 4:**
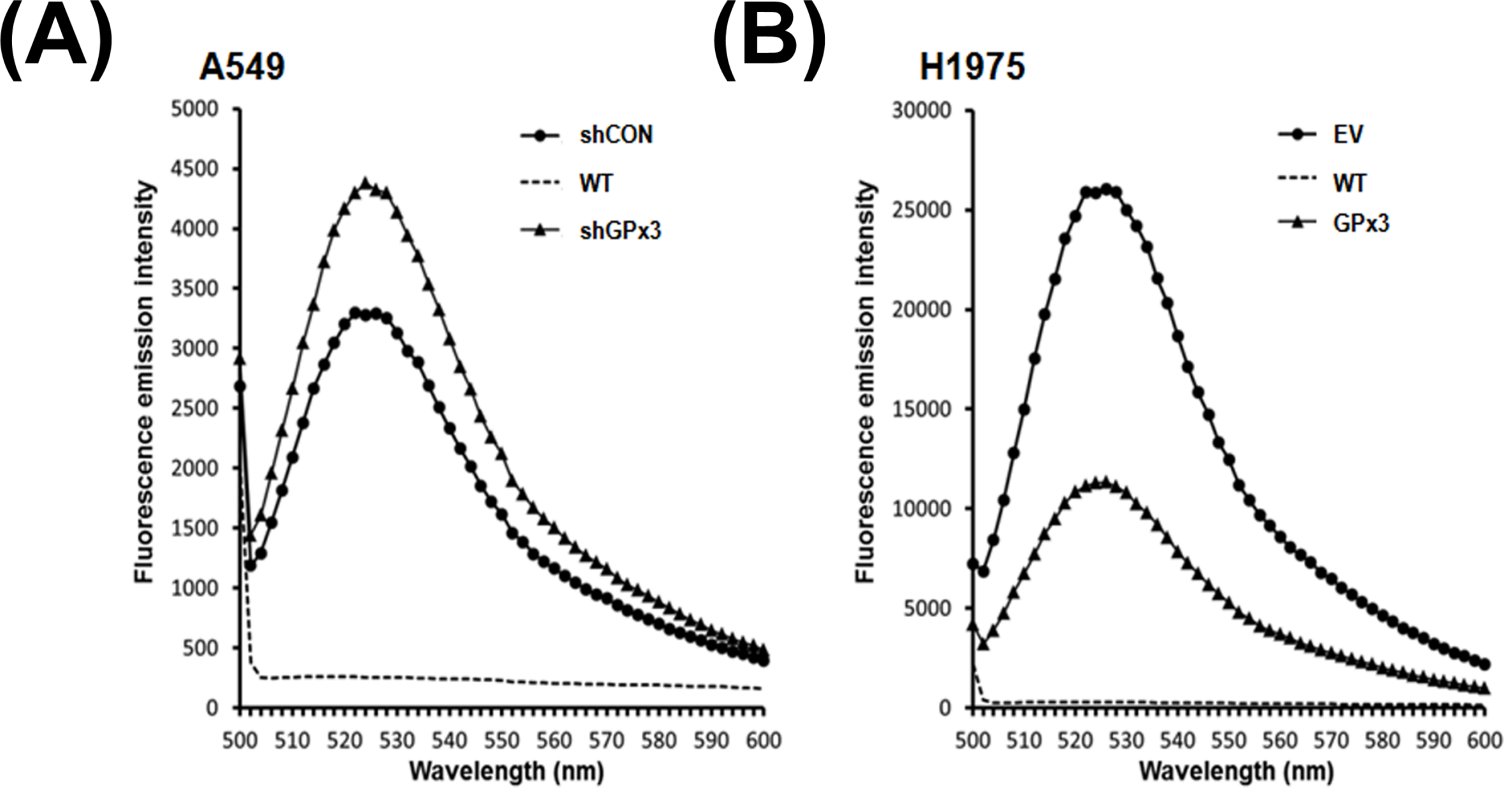
GPx3 inactivates ROS in lung cancer cells. Changes in ROS levels upon oxidative stress are dependent on GPx3 expression. (A) Comparison of ROS levels between A549 lung cancer cells transfected with GPx3 shRNA (shGPx3) and those transfected with control shRNA (shCON). (B) Comparison of ROS levels between H1975 lung cancer cells containing the GPx3 overexpression vector (GPx3) and those containing the empty vector (EV). For normalization, cells transfected with either shCON or EV were used as an internal control. Untransfected cells (A549 control and H1975 control) were used as a negative control. Data are representative of three different experiments.

GPx3 expression is associated with reduced ROS levels in cells [30, 40]. Therefore, we next asked whether GPx3 expression impacts ROS levels in lung cancer cells after serum starvation. Cells expressing low levels of GPx3 [A549 (GPx3 shRNA) and H1975 (EV)] showed increased ROS production. By contrast, cells with high GPx3 expression [A549 cells transfected with control shRNA and H1975 (GPx3) cells] showed significantly reduced ROS production (Fig 4A and 4B). Moreover, we showed that GPx3-dependent ROS inactivation occurs at levels comparable with those induced by H_2_O_2_ at concentrations higher than 100 mM (S1 Fig). This suggests that GPx3 might inhibit tumor progression by regulating the redox balance. A previous report shows that GPx3 binds to p53-induced gene 3 (PIG3), thereby potentiating H_2_O_2_ production and triggering apoptosis [41]. However, this runs counter to the enzymatic (peroxidase) activity of GPx3 in cells. In addition, many studies demonstrate that reduced ROS levels are mediated by overexpression of GPx3 in many types of cancer cell [30, 40].

### GPx3 suppresses proliferation of lung cancer cells

**Fig 5:**
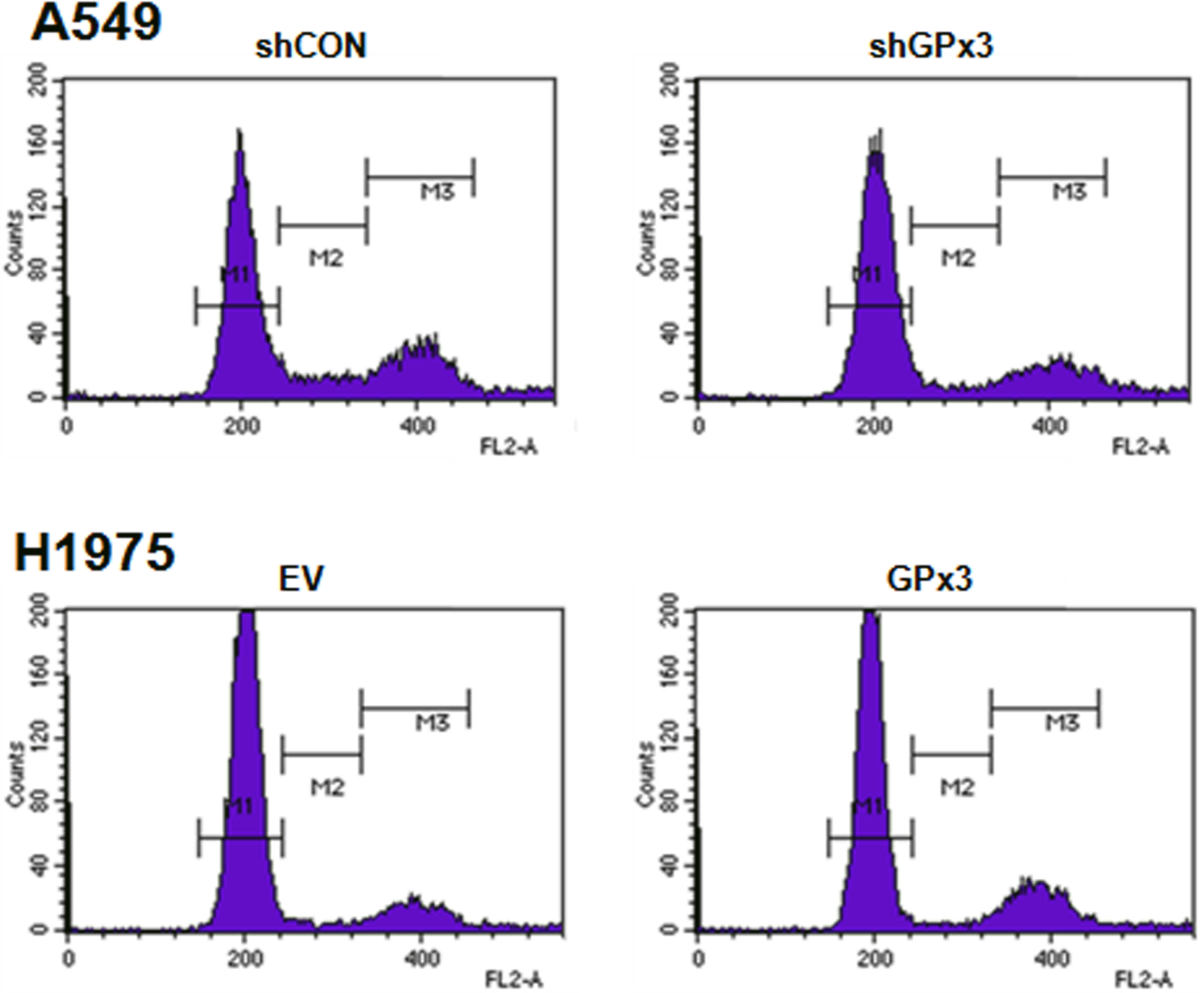
GPx3-mediated cell cycle arrest in lung cancer cells. The anti-proliferative effect of GPx3 expression on the cell cycle was determined by flow cytometry. Comparison of proliferation of A549 lung cancer cells transfected with GPx3 shRNA (shGPx3) (A) and those transfected with control shRNA (shCON) (B) Comparison of the proliferation of H1975 lung cancer cells containing the GPx3 overexpression vector (GPx3) (A) and those containing the empty vector (EV) (B). For normalization, cells transfected with either shCON or EV were used as a control. Data are representative of three independent experiments.

We next examined how GPx3 inhibits the proliferation of lung cancer cells using FACS analysis to measure changes in cell cycle distribution. Serum starvation not only increases ROS levels in cells but also arrests them at G1 phase of the cell cycle [42, 43]. Therefore, we compared the cell cycle distribution of each GPx3 transfectant with that of its corresponding parental control (Fig 5). We found that compared with control cells (cells transfected with empty vector), a greater percentage of GPx3-overexpressing cells (H1975) were in the S and G2/M phases of the cell cycle after transfection with pCI-neo-SECIS::*GPx3* (8% and 16% versus 16% and 38%, respectively). Also, silencing of GPx3 in A549 cells reduced the percentage of cells in the S and G2/M phases when compared with control cells (cells transfected with shRNA) (7% and 17% versus 17% and 40%, respectively). Thus, overexpression of GPx3 in lung cancer cells causes an increased percentage of cells to stall in the S and G2/M phases. It is noteworthy that GPx3 expression in itself did not induce apoptosis of cells subjected to oxidative stress (S1 Fig).

### GPx3 suppresses cyclin B1 expression by inhibition activation of NF-κB

**Fig 6:**
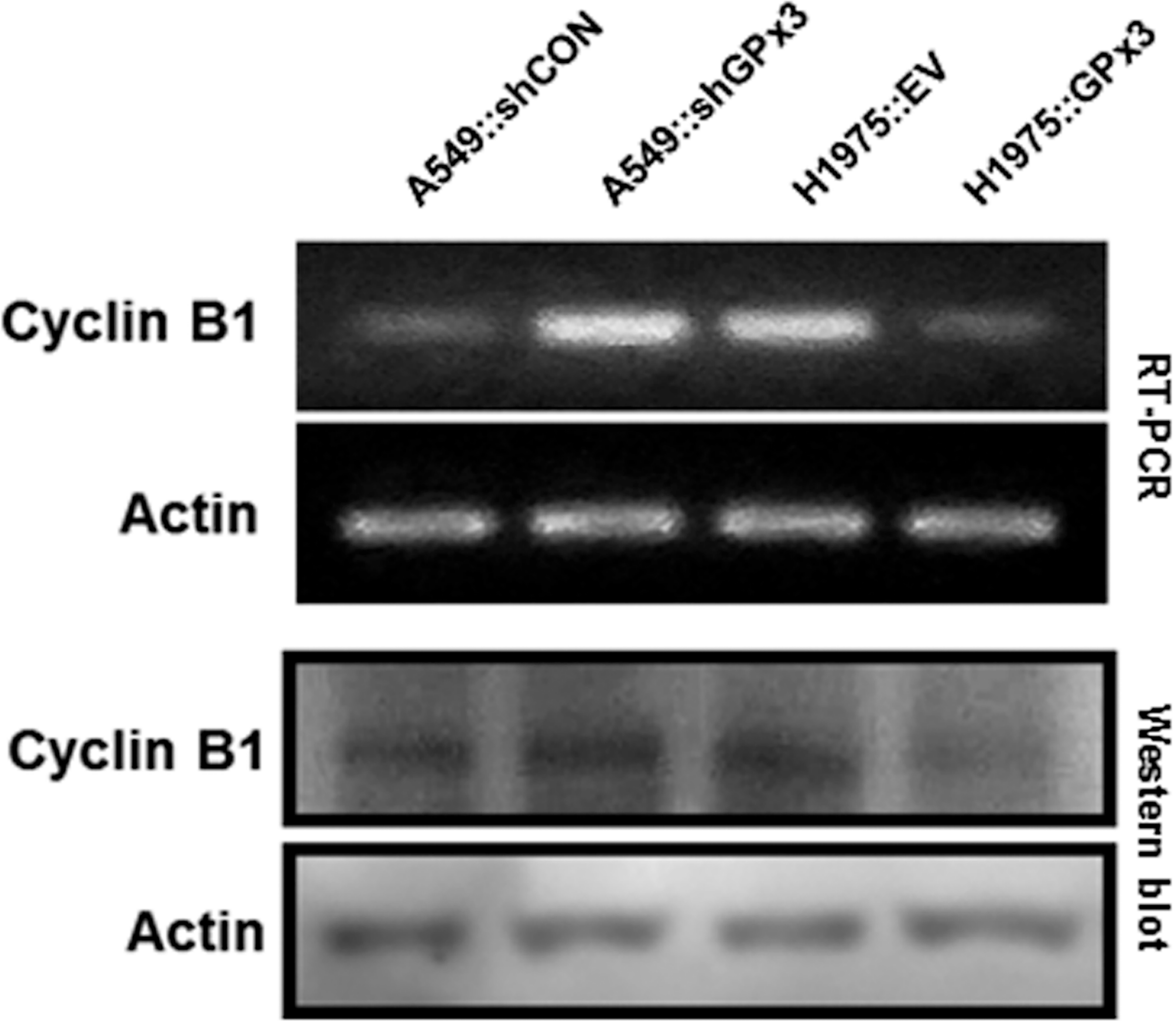
Overexpression of GPx3 down-regulates cyclin B1. GPx3 reduces cyclin B1 expression under conditions of oxidative stress. Changes in expression of cyclin B1 mRNA and protein were measured by RT-PCR and Western blotting. Data are representative of three independent experiments.

**Fig 7:**
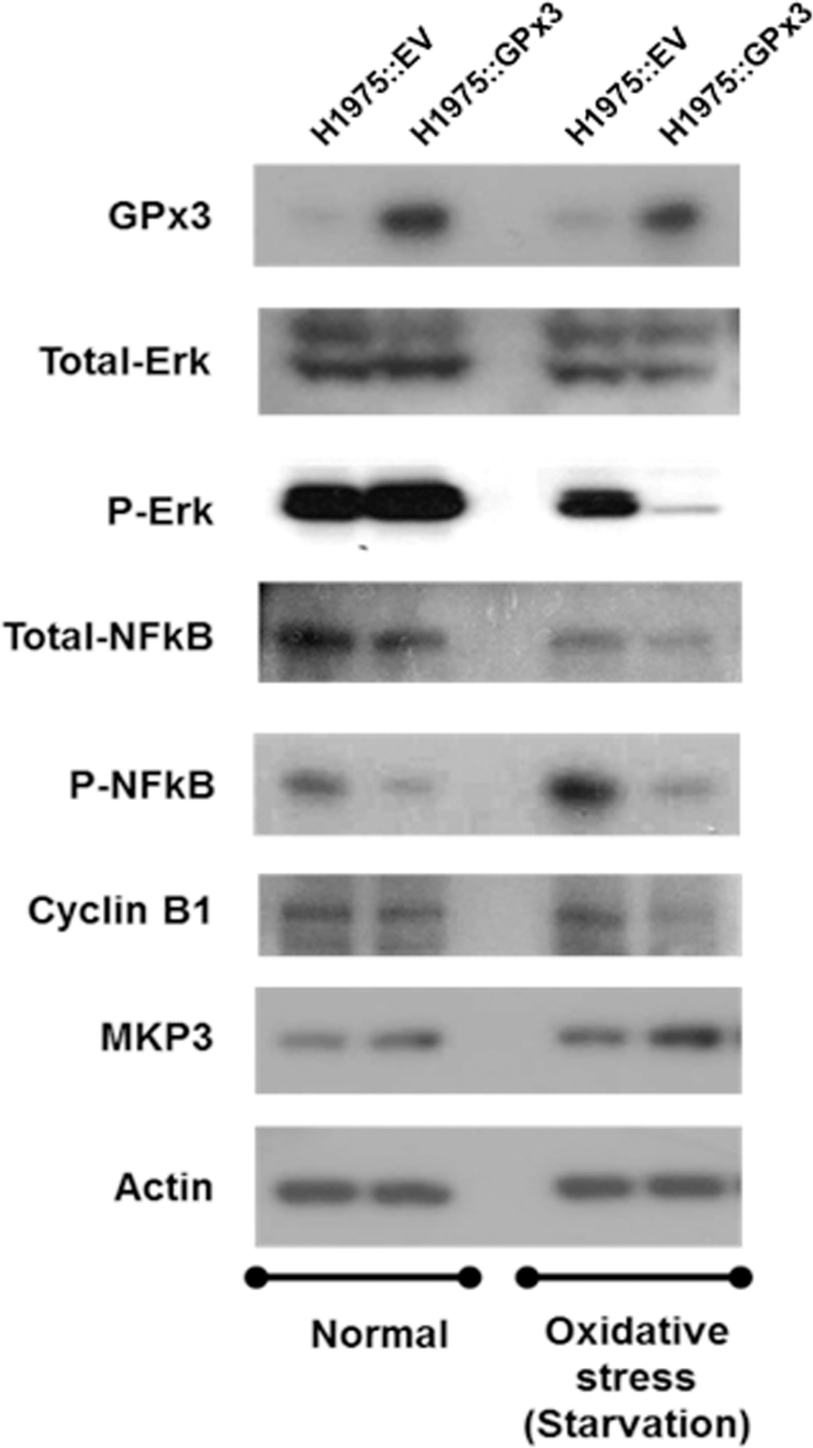
GPx3-mediated regulation of redox signaling pathways has an anti-proliferative effect in lung cancer cells. GPx3 rescues MKP3 expression by protecting it from ROS-mediated degradation under conditions of oxidative stress. GPx3-mediated activation of MKP3 inactivates Erk, leading to reduced NF-κB activation and, ultimately, downregulation of cyclin B1 in lung cancer cells.

Cyclin B1 is an essential regulatory subunit required for transition from G2 phase to mitosis [44]. Moreover, reduced cyclin B1 expression occurs upon extension of G2 phase arrest [45, 46] whereas overexpression reduces the time in G2 phase [47]. Therefore, we next examined whether the level of cyclin B1 expression after oxidative stress is dependent upon GPx3 expression (Fig 6). We found a significant reduction in cyclin B1 levels upon overexpression of GPx3. Ozeki et al. report that cyclin B1 expression is controlled by NF-κB, as the former has an NF-κB binding site in its promoter region [11]. They found that cyclin B1 expression was intimately affected by the efficiency of NF-κB translocation to the nucleus; moreover, ROS inactivation by MnSOD repressed cyclin B1 expression in human breast cancer cells. Therefore, we next investigated whether NF-κB activation (phosphorylation and translocation to the nucleus) depends on GPx3 expression after oxidative stress (Fig 7). Furthermore, we also examined alterations in NF-κB translocation to the nucleus (S2 Fig). We found that GPx3 overexpression significantly reduced nuclear translocation of NF-κB upon oxidative stress, thereby inhibiting cyclin B1 expression in lung cancer cells.

### GPx3 down-regulates extracellular signal-regulated kinase (Erk) by protecting MAP Kinase Phosphatase 3 (MKP3) from ROS

Erk activates specific downstream phosphorylation events during diverse cellular responses such as cell proliferation, growth, differentiation, and survival [48]. Therefore, we examined the influence of GPx3 on Erk expression and activation (phosphorylation) in lung cancer cells subjected to oxidative stress (Fig 7). Overexpression of GPx3 led to a minor change in Erk expression; however, there was significant reduction in Erk phosphorylation. A previous study shows that ROS immediately inactivate and degrade MKP3, which in turn leads to aberrant Erk activation [49]. Moreover, Qi et al. suggest that GPx3 protects MKP3 from degradation by ROS [33]. Therefore, we next examined changes in MKP3 levels in H1975 cells after oxidative stress. We found that MKP3 levels were significantly lower under conditions of oxidative stress (induced either by serum starvation or by exogenous H_2_O_2_) than under normal conditions (S3 Fig). We also observed that although GPx3 protected MKP3 under conditions of oxidative stress, this was not the case under normal conditions. These data suggest that MKP3 in lung cancer cells is degraded immediately upon exposure to ROS generated by oxidative stress. Overexpression of GPx3 rescues the Erk-specific phosphatase activity of MKP3 by inactivating ROS.

## Discussion

We previously reported that plasma GPx3 is a bio-marker of lung cancer as levels in patients with lung cancer are lower than those in normal individuals [32]. Plasma GPx3 is a peroxidase that catalyzes the degradation of H_2_O_2_, organic hydroperoxide, and lipid peroxides by reduced glutathione [21, 31]. The mechanism underlying GPx3 regulation has been well studied. For example, we recently reported the up-regulation of GPx3 via GR activation in lung cancer cells [27]. Many studies report that downregulation of GPx3 in many types of human cancer is caused by promoter hypermethylation [28, 29]. The most important cellular characteristic of GPx3 is its tumor suppressor activity, which has been reported by many groups [28–30]. Previous studies suggest that the tumor suppressor activity of GPx3 is associated with ROS inactivation, which protects cells from genetic mutation and oxidation of proteins involved in carcinogenesis [30]. Moreover, Yu et al. demonstrated that GPx3 suppresses prostate cancer by inhibiting c-Met expression [29]. Recently, Qi et al. reported that GPx3 inhibits invasion of hepatocellular carcinoma cells [33]. In another report, Li et al. demonstrated that CISD2 depletion causes ROS accumulation in lung cancer cells and increases the levels of tumor suppressors EGR1 and GPx3. Furthermore, CISD2 mediated ROS-EGR1-PTEN-AKT signaling causes EMT inhibition in lung cancer cells [34]; however, the authors did not explain the underlying mechanism, although they did show that GPx3 overexpression inhibits cell growth.

Here, we identify the mechanism by which GPx3 inhibits proliferation of lung cancer cells. First, to examine the antioxidant effects of GPx3 on tumor suppression, we used cells subjected to oxidative stress via serum starvation (to induce production of endogenous H_2_O_2_) rather than exposure to exogenous oxidants [35, 36]. We found that serum starvation generated ROS in amounts comparable with those induced by exposure to 100 μM H_2_O_2_ for 10 minutes (S1 Fig). We also found that GPx3 expression after oxidative stress significantly suppressed the proliferation, migration and invasion of lung cancer cells (Fig 3) and specifically arrested cells in the G2/M phase of the cell cycle (Fig 5). However, GPx3 expression had no effect on apoptosis, which is agreement with the findings of Qi et al. [33]. Cyclin B1 is an essential cell cycle component required for transition from G2- to M phase [45, 46, 49]. Two hypotheses have been put forward to explain ROS-mediated cell cycle regulation by cyclin B1 at G2/M phase transition. First, cell division cycle 25 (Cdc25), a cell cycle phosphatase, activates the CDK complex (cyclin B1/Cdk1) required for M phase entry by removing the inhibitory phosphorylation on Cdk1 [50–52]. ROS induce intra-molecular disulfide bond formation (Cys330-Cys377), making the oxidized form susceptible to degradation [53]. Activation of the CDK complex is also inhibited by decreasing Cdc25C levels. According to our results, GPx3 expression immediately reduces ROS level in cells, after which (if the Cdc25 cascade is responsible for GPx3-induced effects) the non-oxidized form of Cdc25 induces M phase entry, resulting in cell proliferation. Another hypothesis proposes that ROS degrade MKP3 via the proteasome/ubiquitination pathway [49]. Activation of the Erk-NF-κB pathway due to MKP3 degradation promotes translocation of NF-κB to the nucleus [33]. Active NF-κB promotes expression of cyclin B1 by binding to the first intron of the cyclin B1 gene [11]. GPx3 and MnSOD inhibit activation of the Erk-NF-κB pathway by protecting MKP3 from ROS; MPK3 then dephosphorylates and inactivates Erk [11, 33]. In fact, we found that oxidative stress induced MKP3 degradation, and that phosphorylation of Erk and NF-κB is dependent on GPx3 expression. Moreover, we observed that reduced NF-κB nuclear translocation occurs after GPx3 expression (S2 Fig) or treatment with PDTC, an NF-κB specific inhibitor, as does reduced cyclin B1 expression (S4 Fig).

**Fig 8:**
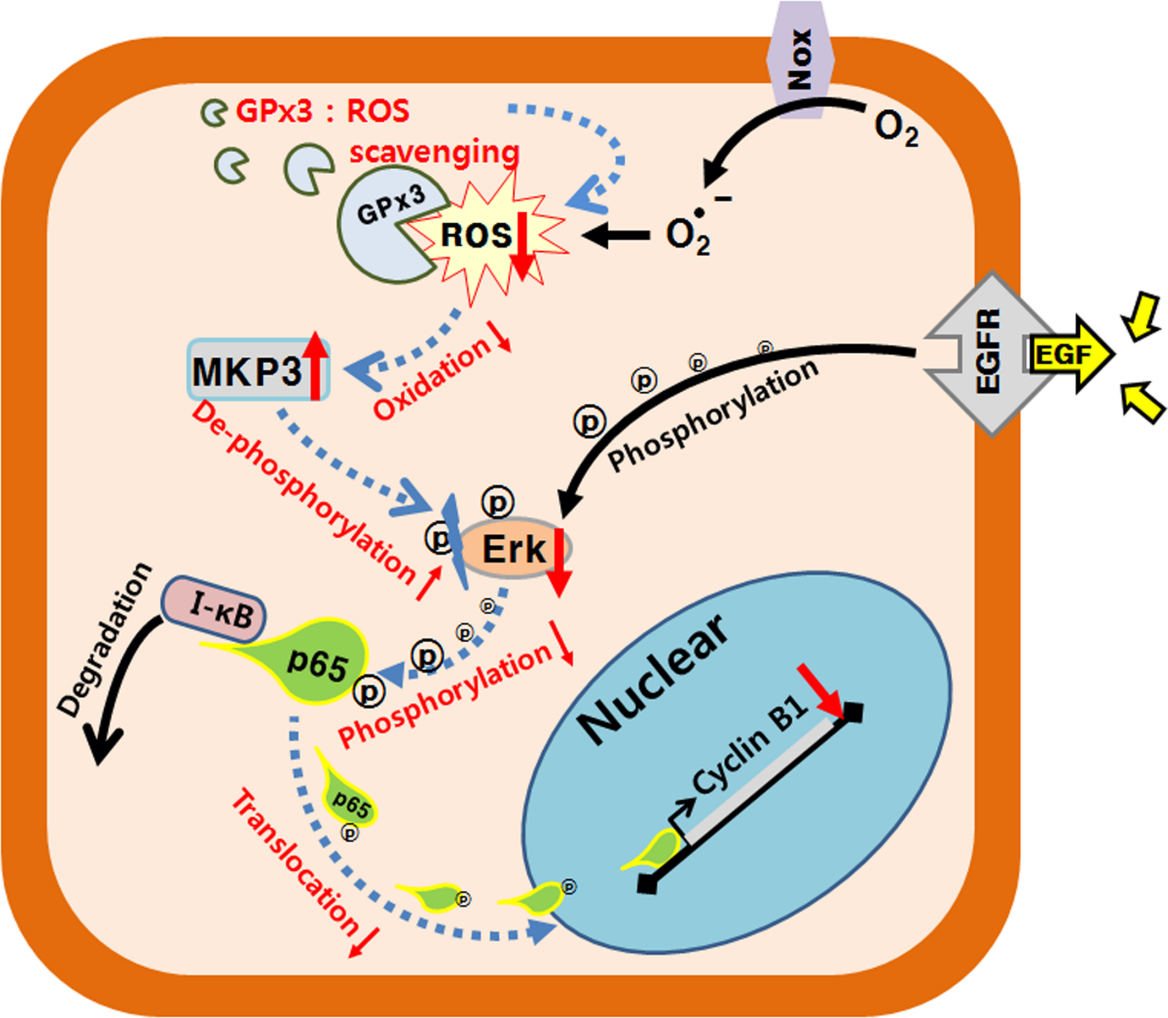
A schematic diagram showing how the anti-oxidant activity of GPx3 could mediate lung cancer suppression. A model of GPx3-mediated redox signaling in the ROS-MKP3-Erk-NF-κB-cyclin B1 pathway in lung cancer cells. Arrows with blue dotted lines represent GPx3-mediated redox signaling under the GPx3 overexpression condition. Text/arrows in black lines represent EGF signaling under the normal condition. Arrows pointing upward, upregulation; arrows pointing downward, downregulation.

In conclusion, the results presented herein demonstrate GPx3 inactivates ROS, thereby suppressing the Erk-NF-κB-cyclin B1 signaling pathway and causing cell cycle arrest at G2/M phase (Fig 8). To the best of our knowledge, this is the first report to identify the mechanism underlying GPx3-mediated inhibition of lung cancer cell proliferation. Further studies are needed to elucidate the network of antioxidant proteins that mediating redox signaling in cancer cells [11, 20, 33].

## Supporting information

S1 Fig GPx3 inactivates exogenous H_2_O_2_ in lung cancer cells. Changes in ROS levels after treatment with 100 μM H_2_O_2_ for 6 h. (A) Comparison of A549 lung cancer cells transfected with GPx3 shRNA (shGPx3) and those transfected with control shRNA (shCON). (B) Comparison of H1975 lung cancer cells containing the GPx3 overexpression vector (GPx3) and those containing the empty vector (EV). Non-stressed cells transfected cells with shCON or EV were used as an internal control. Data are representative of three independent experiments.
(TIF)

S2 Fig GPx3-mediated inhibition of NF-κB translocation to the cell nucleus. GPx3 expression inhibits the translocation of NF-κB in lung cancer cells subjected to oxidative stress.
(TIF)

S3 Fig Exogenous H_2_O_2_ degrades MKP3 in a dose-dependent manner. H1975 cells were exposed to increasing concentrations (0–10 mM) of H_2_O_2_ for 6 h and the levels of MKP3 were measured.
(TIF)

S4 Fig NFkB inhibitor(PDTC)-mediated down-regulation of G2/M signaling. H1975(EV) cells were exposed to 60 μM PDTC for 24 h and the levels of NF-κB and Cyclin B1 were measured.
(TIF)
