# Supplementary figures and images for: GPx3-mediated redox signaling arrests the cell cycle and acts as a tumor suppressor in lung cancer cell lines
Source: PLoS One. 2018 Sep 27;13(9):e0204170. doi: 10.1371/journal.pone.0204170 (PMC6160013; doi:10.1371/journal.pone.0204170)

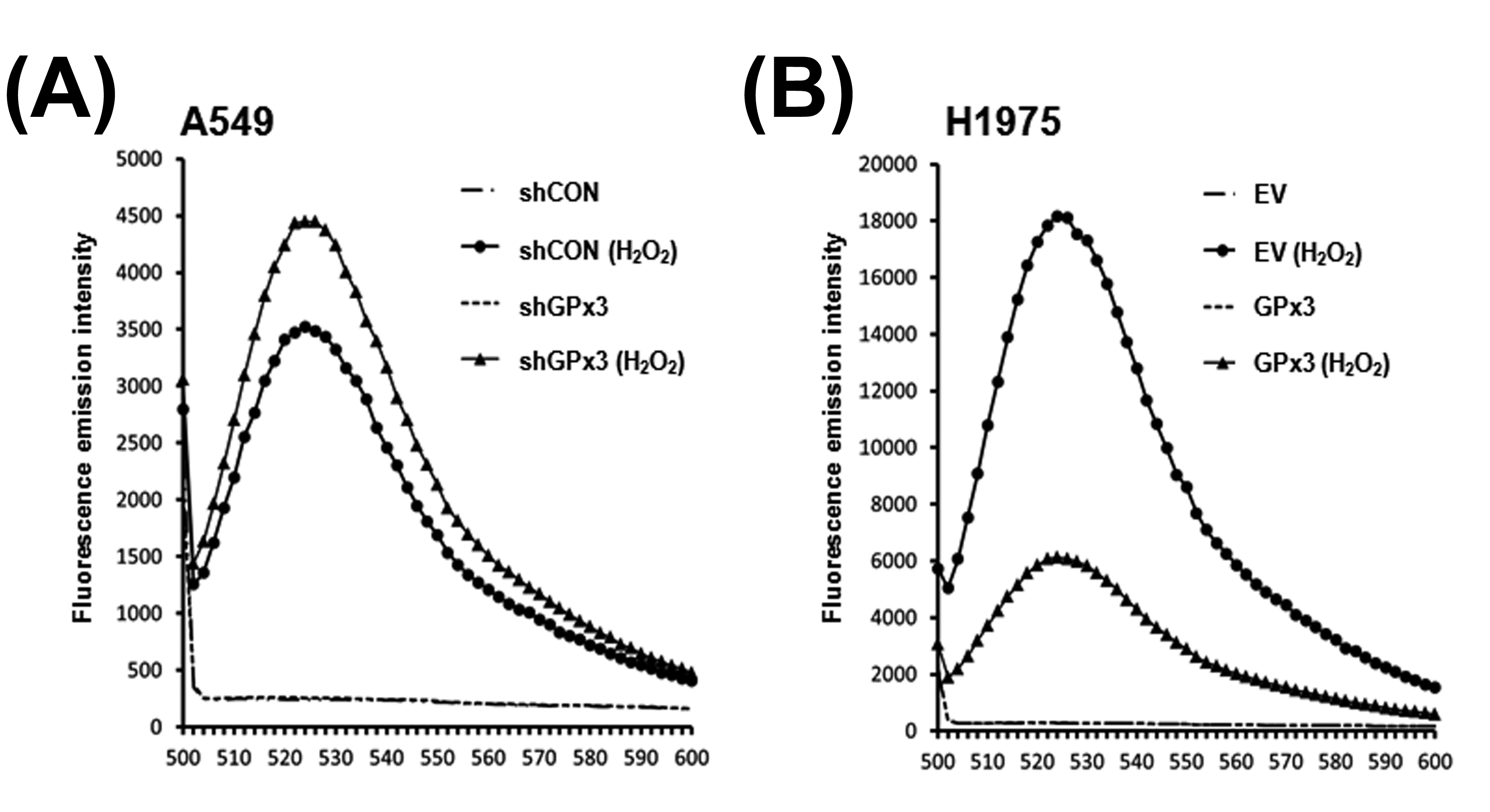

Supplement: S1 Fig — Changes in ROS levels after treatment with 100 μM H2O2 for 6 h. (A) Comparison of A549 lung cancer cells transfected with GPx3 shRNA (shGPx3) and those transfected with control shRNA (shCON). (B) Comparison of H1975 lung cancer cells containing the GPx3 overexpression vector (GPx3) and those containing the empty vector (EV). Non-stressed cells transfected cells with shCON or EV were used as an internal control. Data are representative of three independent experiments. (TIF) [file pone.0204170.s001.tif]

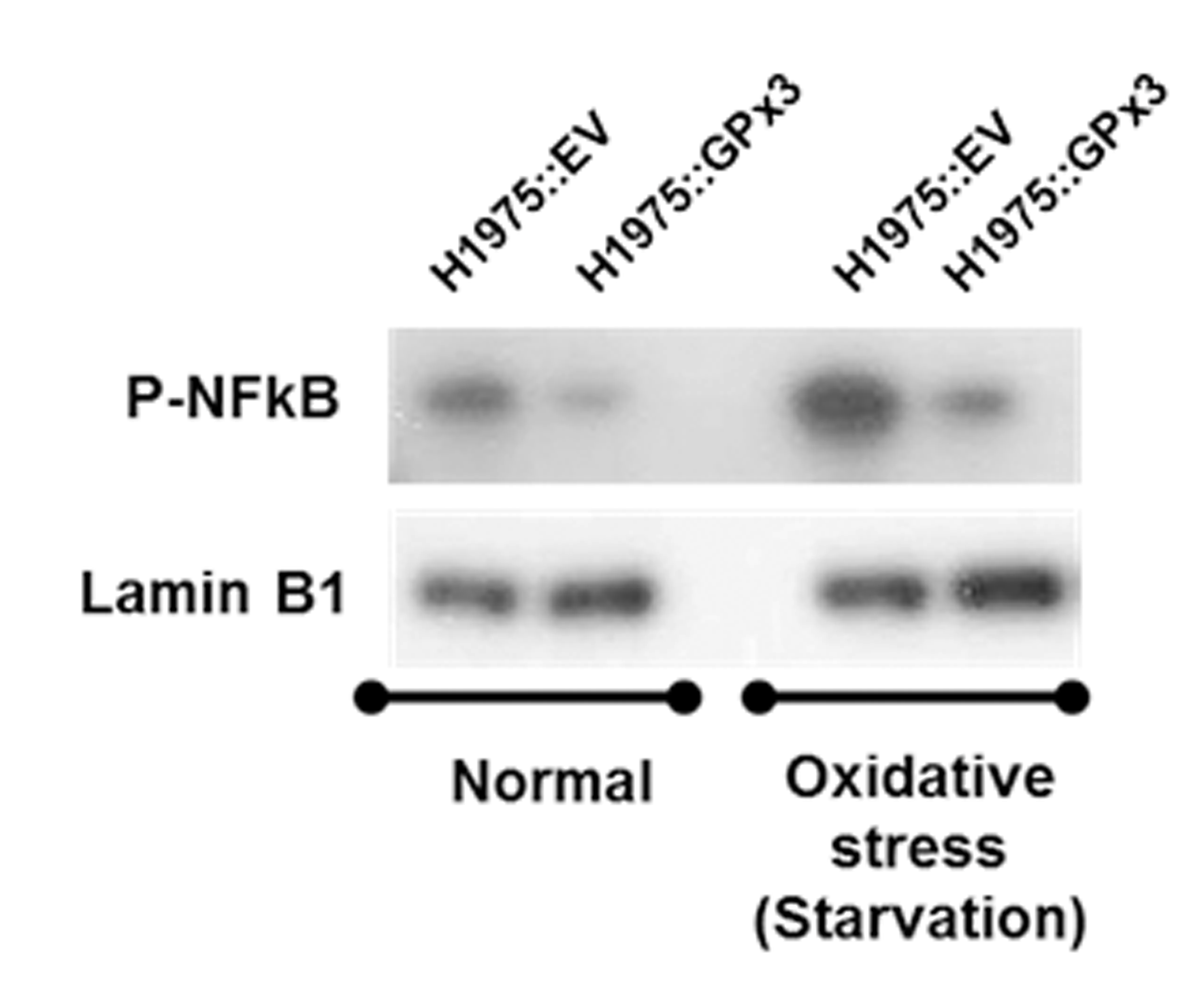

Supplement: S2 Fig — GPx3 expression inhibits the translocation of NF-κB in lung cancer cells subjected to oxidative stress. (TIF) [file pone.0204170.s002.tif]

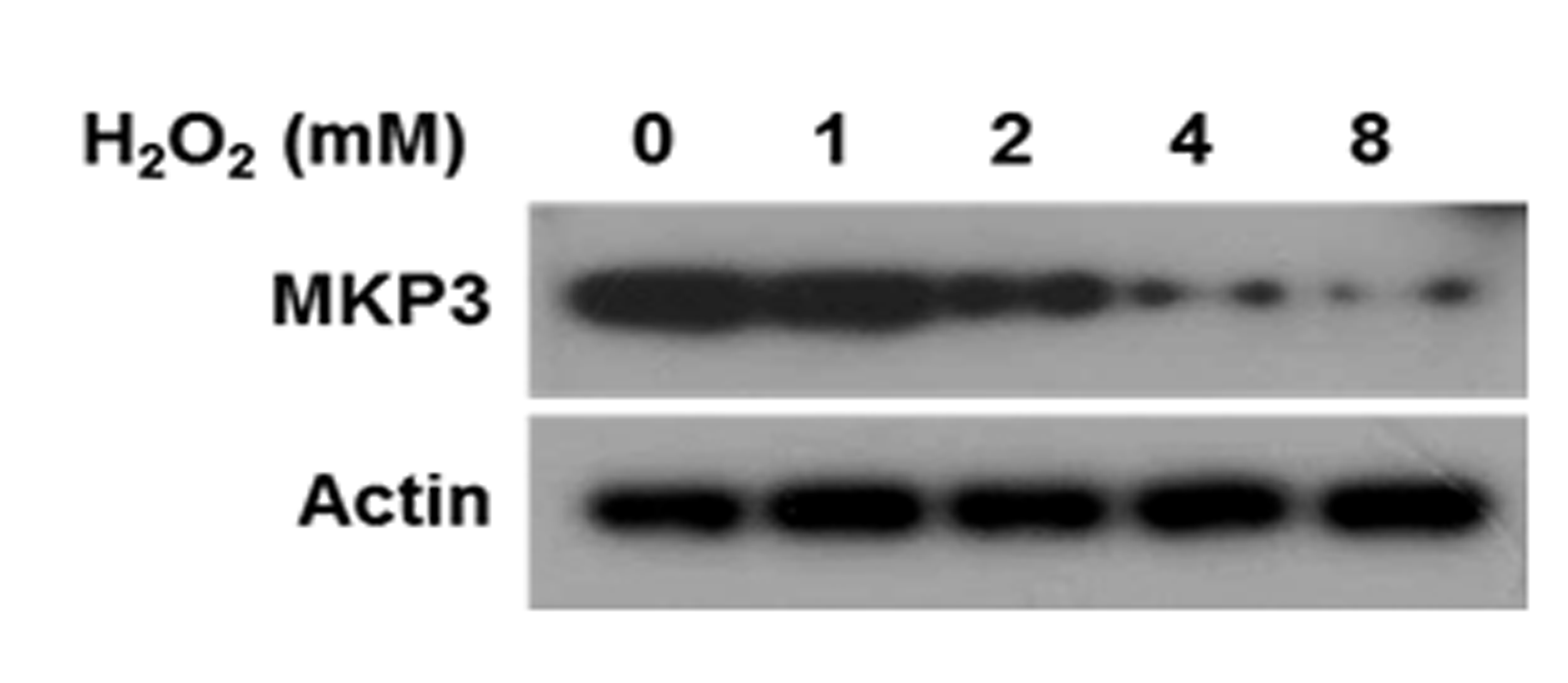

Supplement: S3 Fig — H1975 cells were exposed to increasing concentrations (0–10 mM) of H2O2 for 6 h and the levels of MKP3 were measured. (TIF) [file pone.0204170.s003.tif]

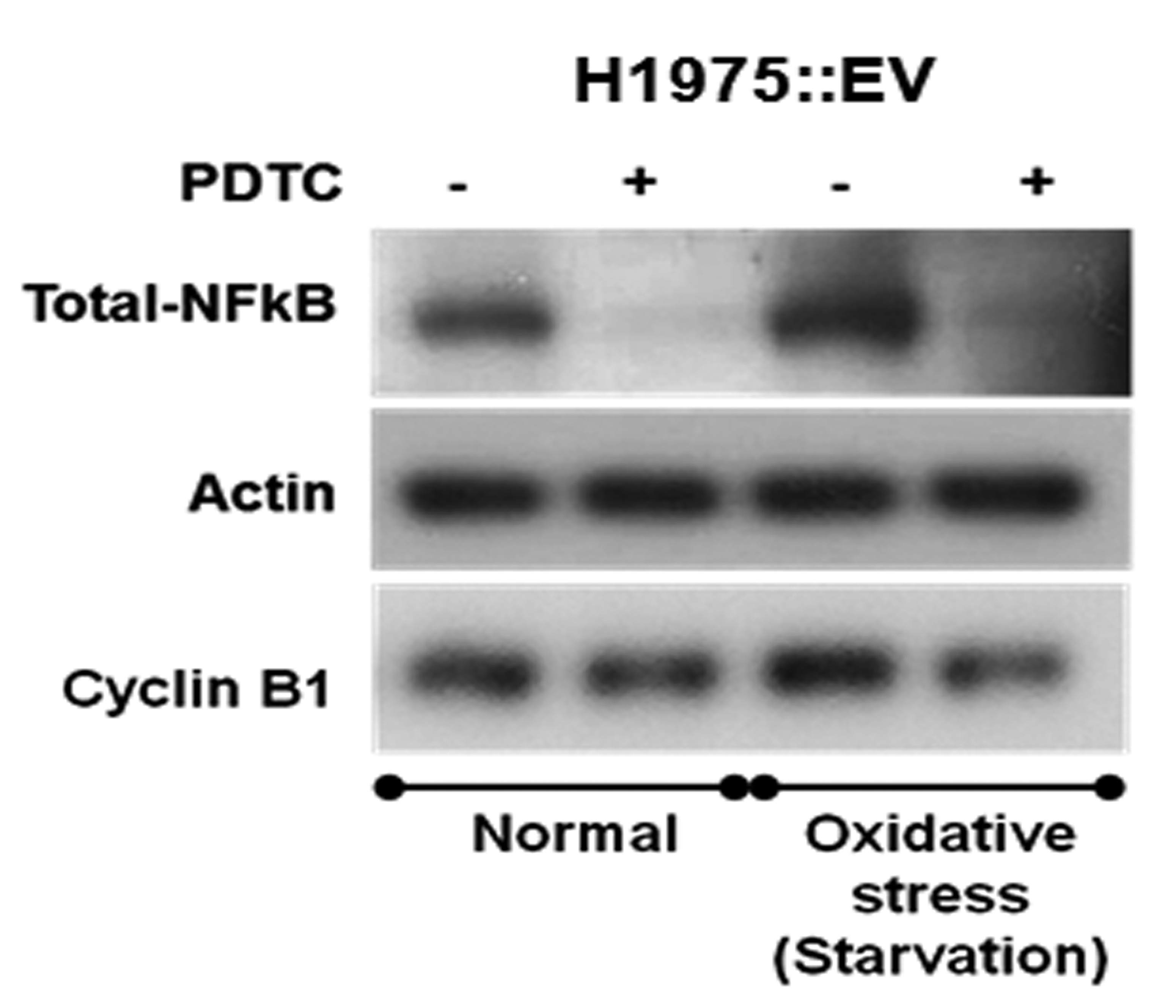

Supplement: S4 Fig — H1975(EV) cells were exposed to 60 μM PDTC for 24 h and the levels of NF-κB and Cyclin B1 were measured. (TIF) [file pone.0204170.s004.tif]
